# Development and validation of a model for the adoption of structured and standardised data recording among healthcare professionals

**DOI:** 10.1186/s12911-018-0640-8

**Published:** 2018-06-28

**Authors:** Erik Joukes, Ronald Cornet, Martine C. de Bruijne, Nicolette F. de Keizer, Ameen Abu-Hanna

**Affiliations:** 1 Department of Medical Informatics, Amsterdam Public Health research institute, Academic Medical Center, University of Amsterdam, P.O. Box 22700, 1100 DE Amsterdam, The Netherlands; 2 Department of Public and Occupational Health, Amsterdam Public Health research institute, VU University Medical Center, Van der Boechorststraat 7, 1081 BT Amsterdam, The Netherlands

**Keywords:** Electronic health records, Adoption, Intention, Structured, Standardised, Recording, Structural equation modelling

## Abstract

**Background:**

Healthcare professionals provide care to patients and during that process, record large quantities of data in patient records. Data in an Electronic Health Record should ideally be recorded once and be reusable within the care process as well as for secondary purposes. A common approach to realise this is to let healthcare providers record data in a standardised and structured way at the point of care. Currently, it is not clear to what extent this structured and standardised recording has been adopted by healthcare professionals and what barriers to their adoption exist. Therefore, we developed and validated a multivariable model to capture the concepts underlying the adoption of structured and standardised recording among healthcare professionals.

**Methods:**

Based on separate models from the literature we developed a new theoretical model describing the underlying concepts of the adoption of structured and standardised recording. Using a questionnaire built upon this model we gathered data to perform a summative validation of our model. Validation was done through partial least squares structural equation modelling (PLS-SEM). The quality of both levels defined in PLS-SEM analysis, i.e., the measurement model and the structural model, were assessed on performance measures defined in literature.

**Results:**

The theoretical model we developed consists of 29 concepts related to information systems as well as organisational factors and personal beliefs. Based on these concepts, 59 statements with a 5 point Likert-scale (fully disagree to fully agree) were specified in the questionnaire. We received 3584 responses. The validation shows our model is supported to a large extent by the questionnaire data. Intention to record in a structured and standardised way emerged as a significant factor of reported behaviour (β = 0.305, *p* < 0.001). This intention is influenced most by attitude (β = 0.512, *p* < 0.001).

**Conclusions:**

This model can be used to measure the perceived level of adoption of structured and standardised recording among healthcare professionals and further improve knowledge on the barriers and facilitators of this adoption.

**Electronic supplementary material:**

The online version of this article (10.1186/s12911-018-0640-8) contains supplementary material, which is available to authorized users.

## Background

Healthcare professionals provide care to patients and record large quantities of data in patient records during that process. These data are used in daily care practice as records of the history of a patient, parts of the thought process of the physician, and the planned course of treatment. These data are used to make informed decisions about diagnosis and treatment. These data are increasingly recorded digitally in electronic health records (EHRs). These systems, and their underlying databases, enable storage and easy retrieval of data. By storing the data in an electronic form, the possibilities of data reuse increase. The data can be reused for other purposes such as decision support, generation of discharge letters, scientific research, management information, quality assurance through auditing registries, and reimbursement. However, for data to be fully reusable they have to be stored structured and standardised. A common approach to realise this is to let healthcare providers record data in a standardised and structured way at the point of care.

The main focus of our study is recording at the point of care of structured and standardised data that are reusable within the care process as well as for secondary purposes. This means that healthcare professionals must record data in an Electronic Health Record once, in a standardised and structured way by using structured forms and coding systems. This specific method of recording differs from the way of working that numerous physicians have been used to for decades, using free text for precisely recording the patient status, combined with sometimes multiple ways of coding for research. This means that structured and standardised data recording is not automatically and fully adopted by healthcare professionals. In addition, the actual data recording might take more time than current working procedures. The efficiency effect of reusing data is not always clear to the physicians, and they have concerns about a higher recording burden [1]. An additional barrier may be that physicians who record the data are not always the ones benefitting from the profits of structured and standardised data recording. For example, physicians might require more time to record in a structured manner, while administrative staff benefits using the data for financial reimbursement or management purposes.

Currently, it is not clear to what extent structured and standardised data recording has been adopted by healthcare professionals. For the management of hospitals the largest impediments for this adoption are unclear. Therefore, in this study, we aim to develop a multivariable model to capture the interrelating concepts underlying the adoption of structured and standardised data recording among healthcare professionals. The model includes concepts related to information systems as well as organisational factors and personal beliefs and can be used to identify those concepts relevant to the adoption of structured and standardised recording and barriers that currently limit the adoption. The results of our model should further our understanding of the underlying theory pertaining to structured and standardised data recording. Additionally, this might help hospital management and national coordinating organizations to improve the adoption by working on identified barriers, thereby using the limited available resources of these organizations to solve the most limiting factors holding back the adoption.

To evaluate the validity of our theoretical model we performed a summative evaluation. The results of this evaluation indicate to what extent the model is supported by the collected data obtained by questionnaires. Additionally, this evaluation can give leads to where future research can update and improve our theoretical model.

## Methods

Our method consists of four steps. First, we developed the model based on other validated models from the literature. A number of models have described the usage intention or acceptance of a specific system by the users, or the system’s success [2–5]. Our main outcome is, however, not the intention to use a system but the intention to record data in a certain way (i.e. structured and standardised). Therefore, we need to develop and validate a new model that can be used to measure those healthcare professionals’ intentions. Second, based on this model we created a questionnaire. Third, we used our questionnaire to collect data from healthcare professionals. Finally, we use partial least squares structural equation modelling to empirically validate our model using data we collected in the third step. Further details on all four steps are described below.

### Development of the theoretical model

The outcome of our model will be the (self-reported) adoption of structured and standardised data recording. We performed an exploratory literature search to identify models that describe the acceptance of electronic healthcare systems and human-computer interactions. From those models we selected two models [6, 7] that were relevant to our goal. The model of Wixom and Todd [7] describes an integrated model combining user satisfaction and technology acceptance. The model of Hsieh [6] targets the acceptance of electronic medical records exchange. Since these two models are both based on the Technology Acceptance Model (TAM), we were able to link them on matching concepts (perceived usefulness, perceived ease of use, attitude, and intention). Both models have been validated with structural equation modelling [6, 7]. Based on other literature [8] we added specific concepts that the models were lacking, addressing the goal of our model.

### Questionnaire development and data collection

For each theoretical concept in our model, we specified at least one concrete question that covers the concept. Together with demographic questions this set of questions were presented in an online questionnaire. This questionnaire was sent to healthcare professionals from seven out of the eight Dutch university hospitals. All professions working with patient data or the EHR were included (e.g. physicians, nurses, researchers). In five hospitals, all personnel working with patient data were included, in two hospitals a random selection of 1000 people were included. Data were collected between May and November 2015. The first question of the questionnaire was whether the respondent was an active or passive user of the questionnaire. Passive users only read data from the EHR, whereas active users also record data in the system. The active users received the full questionnaire, the passive users only a selection of relevant questions. Only the active user respondents were included in the current analysis.

### Model validation

To validate our model we performed structural equation modelling (SEM) using the partial least squares (PLS) method [9]. SEM is a group of multivariate techniques combining aspects of factor analysis and regression where relationships among observed variables (the questions in the questionnaire) and latent variables (the concepts in the model), as well as among latent variables are analysed [9]. The PLS variant of SEM is especially suitable for models with a high number of latent and observed variables. Additionally, PLS does not require the data to be normally distributed. The technique is used both within [6, 10] and outside [7] of the healthcare domain.

**Fig. 1:**
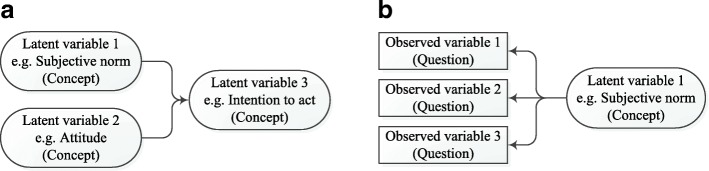
**a** Structural model - showing the relation between three latent variables (concepts from our theoretical model). **b** Measurement model - showing the relation between three observed variables (questions from our questionnaire) and one latent variable (concept from the theoretical model).

In SEM the distinction is made between the measurement model and the structural model (see Fig. 1a and b). The structural model was obtained from the development of the theoretical model. This structural model (Fig. 1a) shows the latent variables and their interrelations as we have defined them a priori. These latent variables are the concepts of our theoretical model which are not measured directly by the questions in the questionnaire.

Each question in our questionnaire, called an observed variable, reflects an aspect of one of the latent variables in the model. In the measurement model (Fig. 1b) the observed variables are linked to the latent variables. This model indicates which observed variables are related to which latent variables. For example, the questions ‘format1’ (corresponding to the statement “The format of the patient record is clear”) and ‘format2’ (“Because of clear formatting, data in the patient record can easily be recognised”) are observed variables referring to format of the data. These are linked to ‘format’, the corresponding latent variable, in the measurement model. The latent variable ‘format’ is linked to the other latent variable ‘information satisfaction’ through the structural model. All the latent variables in our model are reflective (rather than formative), indicating the assumption that the latent variable is responsible for the variability in the observed variables.

We will separately validate the measurement model and the structural model. Validating the measurement model will show whether we actually measure what we want to measure within each concept. For this validation we determined the performance measures as described by Hair et al. [9] and listed in Table 1. The criteria for the validation of the measurement model are not applicable to single-item concepts [9]. Therefore we can only calculate the measures for latent variables that had more than one observed variable.

**Table 1:** Used performance measures and targets to validate the measurement model from Hair et al. [9]

Type of validation	Measure	Target
internal consistency / composite reliability	Dillon Goldstein’s rho (alternatives are Cronbach’s alpha and eigenvalues)	> 0.60 are acceptable in exploratory research
indicator reliability	outer loadings	> 0.708
convergent validity	Average Variance Extracted (AVE)	> 0.5
discriminant validity	A) outer loadings	A) the outer loading of an observed variable on its concept is higher than its cross loadings with other concepts
	B) Fornell-Larcker criterion	B) the square root of the AVE of a concept should be higher than its correlations with all other concepts

The validation of the structural model based on the data that we collected will show whether our a priori defined model is valid. In this step, we evaluated: the coefficients of determination (R^2^) and the size and relevance of the path coefficients.

We used the statistical environment R (version 3.3.1) [11] with the plspm package version 0.4.7 [12]. To adjust for the missing values in our dataset we used stochastic multiple imputation methods from the mice package to create five datasets without missing values. All analyses were performed on one dataset. To determine the effect of this imputation we performed a sensitivity analysis by repeating all analyses on the four additional imputed datasets. We compared the results of the different analyses.

Different types of healthcare providers have different ways of interacting with patient data and EHRs. This means that the performance of the model might be different if we use data of a subgroup based on a specific type of healthcare provider. Therefore in addition to using the full dataset we repeated the model validation using two subsets of the data: data of either medical specialists or nurses, as these are the largest groups of healthcare providers that actively use the EHR. We compared the performance measures for these two subgroups with those of the overall model and evaluated the performance of the two additional models based on the same targets as listed in Table 1. The latter indicates whether the final conclusion concerning the performance of our model would be different when it is based on a subgroup of healthcare providers.

The study design was submitted to the ethics committee of the VU University Medical Center Amsterdam, and was exempt from review (reference 2015.185).

## Results

### Development of the theoretical model

**Fig. 2:**
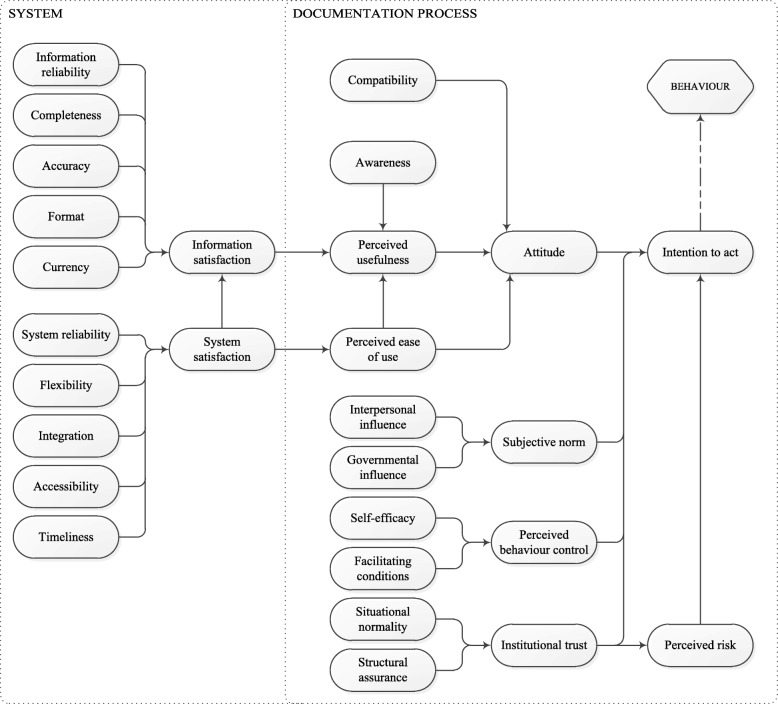
Our proposed theoretical model consisting of 29 concepts (i.e. latent variables)

Figure 2 shows the proposed theoretical model based on the literature. All other hypothesised relations, based on the underlying validated models [6, 7], are depicted therein. For example; system satisfaction influences perceived ease of use and information satisfaction. Table 2 provides the origin and a description of all concepts.

**Table 2:** All concepts in our model, including origin and explanation of each concept

Model concepts	Wixom and Todd [7]	Hsieh [6]	This study	Explanation
Information reliability			X	Whether the information in the EHR is reliable
Completeness	X			Whether the information in the EHR is complete
Accuracy	X			Whether the information in the EHR is accurate
Format	X			Whether the information in the EHR is in an understandable format
Currency	X			Whether the information in the EHR is up to date
System reliability	X			Whether the user can trust that the EHR works
Flexibility	X			Whether the user can use the EHR flexibly in different situations
Integration	X			Whether the user needs to open multiple computer programs to gather all information on patients
Accessibility	X			Whether the user can access the patient data in every place in the organization
Timeliness	X			Whether the system responds to user input in a timely manner
System satisfaction	X			The overall opinion of the user on the quality of the EHR
Compatibility		X		Whether the EHR supports the work processes of the user
Awareness			X	Whether the user knows why it is important that their data are recorded correctly
Perceived ease of use	X	X		The overall opinion of the user on the usability of the EHR
Information satisfaction	X			Whether the user is satisfied with the information that the EHR provides
Perceived usefulness	X	X		Whether the EHR aids in the user’s daily work
Attitude	X	X		What the user thinks of structured and standardised recording
Interpersonal influence		X		Whether the supervisor promotes correct recording
Governmental influence		X		Whether the government (i.e. the inspectorate) promotes correct recording
Subjective norm		X		Whether the user records correctly because colleagues expect this
Self-efficacy		X		Whether the user is capable of correct recording
Facilitating conditions		X		Whether there is enough time to record data correctly
Perceived behavioural control		X		Whether it is within the user’s control to record data correctly
Situational normality		X		Whether it is normal in the organisation to record correctly
Structural assurance		X		Whether the organisation ensures that data are stored safely and cannot be lost
Institutional trust		X		Whether the user trusts that the organisation stores the records safely
Perceived risk		X		Whether the reuse of data can harm the patients’ privacy and or safety
Intention to act	X	X		Whether the user wants to record data structured and standardised and wants to reuse data
Behaviour			X	A number of facets that indicate whether the user is already recording structured and standardised data

The main outcome of our theoretical model is the self-reported behaviour of care professionals, i.e. whether they report to have adopted structured and standardised data recording. Behaviour is influenced by the professional’s intention to act. This intention is based on attitude, subjective norm, perceived behavioural control, institutional trust, and perceived risk. All concepts on the right-hand side of the model, are related to working processes and human attitudes. The concepts influencing attitude describe the professional’s knowledge of structured and standardised data recording and whether they think this way of working is usable, useful, and aligned with their own working processes. The items in the lower right quadrant describe the environmental factors such as the promotion of structured and standardised data recording by supervisors and colleagues, the level of control the professional has, and the perceived risks that the work processes might pose. Improvements in this part of the model need to come from changing the way people perceive their working environment, their work processes, and structured and standardised data recording.

The concepts in the model on the left-hand side of the diagram are all related to the documentation system in place in the organization, in most cases an EHR. These are the concepts that can be influenced by changing aspects of the EHR itself. The items information reliability (from [8]), completeness, accuracy, format, and currency, all indicate separate facets of information quality. They all describe a specific aspect of the stored data or information in an EHR that influences whether the users of the system trust the data (reliability, accuracy, currency) and whether they can actually understand and work with the data (completeness, format). All these items influence whether the user is satisfied with the information that is presented (information satisfaction). The concepts system reliability, flexibility, integration, accessibility, and timeliness represent aspects of system satisfaction. They influence the opinion of the EHR users on the quality of the system.

Finally, we removed two concepts from the Wixom and Todd model (information quality and system quality). For these two items, the questions in our questionnaire were too similar to those that belong to the items information satisfaction and system satisfaction.

### Questionnaire development and data collection

The questionnaire included 59 questions based on all 29 concepts of our model, supplemented with 17 questions on demographic data. Additional file 1 shows an English translation of the original Dutch version of the 59 questions based on the model. We received responses to our questionnaire from 5011 participants of which only the 3584 active users were included in the analyses described in this paper. The demographics of our respondents are summarised in Table 3. The number of missing values was between 6 and 57% (IQR 22–33%) per variable. For more detailed information on missing data see Additional file 2: Table S1.

**Table 3:** Demographics of the included respondents

	n (%)
Total respondents	3584 (100)
Gender	
Male	889 (25)
Female	2413 (67)
Age	
< 30	461 (13)
30–39	879 (25)
40–49	743 (21)
50–59	868 (24)
> =60	270 (8)
Function	
Analytical staff	57 (2)
Clinical (co-)care provider	336 (9)
Medical support staff	223 (6)
Management	90 (3)
Medical specialists	856 (24)
Administrative staff	247 (7)
Nurses	1358 (38)
Scientific research	251 (7)
Other	265 (7)

### Model validation

#### The measurement model

The results of the validation of the measurement model are listed in Tables 4 and 5, and Additional file 2.

**Table 4:** Composite reliability measures of latent variables with more than one observed variable

	number of observed variables	Dillon-Goldstein’s rho
Attitude	4	0.620
Information reliability	4	0.711
Awareness	3	0.730
Perceived usefulness	5	0.766
Integration	2	0.782
Structural assurance	2	0.802
Behaviour	11	0.804
Accuracy	2	0.825
Perceived risk	2	0.831
Intention to act	2	0.831
Perceived ease of use	3	0.866
Format	2	0.925

Latent variables not mentioned in this table have only one observed variable and therefore no scores on these measures

**Table 5:** All observed variables, their latent variable, and their loadings

Observed variable	Latent variable	loading
InformationReliability1	information reliability	**0.719**
InformationReliability2	information reliability	0.461
InformationReliability3	information reliability	0.540
InformationReliability4	information reliability	0.703
Accuracy1	accuracy	**0.850**
Accuracy2	accuracy	**0.825**
Format1	format	**0.930**
Format2	format	**0.926**
Integration1	integration	**0.750**
Integration2	integration	**0.847**
Awareness1	awareness	**0.746**
Awareness2	awareness	0.534
Awareness3	awareness	**0.775**
PerceivedEaseOfUse1	perceived ease of use	**0.804**
PerceivedEaseOfUse2	perceived ease of use	**0.865**
PerceivedEaseOfUse3	perceived ease of use	**0.808**
PerceivedUsefulness1	perceived usefulness	0.576
PerceivedUsefulness2	perceived usefulness	**0.800**
PerceivedUsefulness3	perceived usefulness	**0.774**
PerceivedUsefulness4	perceived usefulness	**0.817**
PerceivedUsefulness5	perceived usefulness	0.090
Attitude1	attitude	0.297
Attitude2	attitude	**0.737**
Attitude3	attitude	0.554
Attitude4	attitude	0.689
StructuralAssurance1	structural assurance	**0.737**
StructuralAssurance2	structural assurance	**0.886**
PerceivedRisk1	perceived risk	**0.943**
PerceivedRisk2	perceived risk	0.701
IntentionToAct1	intention to act	**0.750**
IntentionToAct2	intention to act	**0.917**
Behaviour1	behaviour	−0.034
Behaviour2	behaviour	**0.721**
Behaviour3	behaviour	0.689
Behaviour4	behaviour	**0.731**
Behaviour5	behaviour	0.446
Behaviour6	behaviour	0.340
Behaviour7	behaviour	0.240
Behaviour8	behaviour	0.206
Behaviour9	behaviour	0.536
Behaviour10	behaviour	0.623
Behaviour11	behaviour	0.356

Loadings in bold cells satisfy the prescribed threshold (> 0.708). Each observed variable is a question in our questionnaire, the actual questions are available in Additional file 1

First, we evaluated the composite reliability by calculating the Dillon Goldstein’s rho. All relevant latent variables had a Dillon Goldstein’s rho of more than 0.7, apart from attitude which had a score of 0.62. Hence, all these scores were above the limit of 0.6 suggested for indicating composite reliability. Evaluation of the Cronbach’s alpha and eigenvalues showed qualitatively similar results.

To estimate the indicator reliability, we calculated the loadings of the observed variables on the latent variables. In six of the 12 blocks of latent variables all loadings were >  0.708 (accuracy, format, integration, intention to act, perceived ease of use, and structural assurance). Although the other six blocks (attitude, awareness, behaviour, information reliability, perceived risk, and perceived usefulness) had at least one observed variable that is > 0.708, one or more loadings in these blocks were < 0.708, see Table 5. These loadings varied from − 0.034 to 0.703. Especially behaviour had a number of very low loadings.

The convergent validity is based on the Average Variance Extracted (AVE) of the concepts. In Table 6 these AVEs are reported. Of the 12 concepts that have multiple indicators, seven had an AVE of > 0.5. The other five concepts (information reliability, awareness, perceived usefulness, attitude, and behaviour) had AVEs ranging from 0.250 (behaviour) to 0.481 (awareness).

**Table 6:** Latent variables, mean, sd, and Average Variance Extracted (AVE)

Latent variable	mean	sd	AVE
information reliability	3.74	0.63	0.379
completeness	3.54	1.02	1
accuracy	3.44	0.74	0.701
format	3.12	0.97	0.861
currency	3.52	0.90	1
system reliability	3.47	0.90	1
flexibility	3.23	0.97	1
integration	3.00	0.91	0.640
accessibility	3.67	1.07	1
timeliness	3.15	1.05	1
system satisfaction	2.96	1.03	1
compatibility	3.66	0.87	1
awareness	3.60	0.60	0.481
perceived ease of use	2.99	0.91	0.683
information satisfaction	3.27	0.88	1
perceived usefulness	2.93	0.97	0.449
attitude	3.90	0.51	0.353
interpersonal influence	3.43	0.94	1
governmental influence	3.21	0.89	1
subjective norm	3.62	0.88	1
self-efficacy	3.78	0.84	1
facilitating conditions	2.79	1.02	1
perceived behavioural control	3.63	0.88	1
situational normality	3.50	0.91	1
structural assurance	3.49	0.73	0.664
institutional trust	3.97	0.71	1
perceived risk	2.84	0.75	0.690
intention to act	4.04	0.59	0.701
behaviour	3.42	0.56	0.250

Discriminant validity is based on the cross loadings of all indicators and concepts that are depicted in Additional file 2: Table S2. It shows that five of the 59 indicators had a cross loading that is higher than the loading on its own concept. Three were situated in the behaviour block, one in awareness, and one in perceived usefulness. The difference between the loadings and cross loadings ranged from 0.325 to 0.035. Additionally, the square root of the AVE and the inter-concept correlations are shown in Additional file 2: Table S3. It shows that the square roots of all AVEs were higher than the inter-concept correlations (Fornell-Larcker criterion).

#### The structural model

**Fig. 3:**
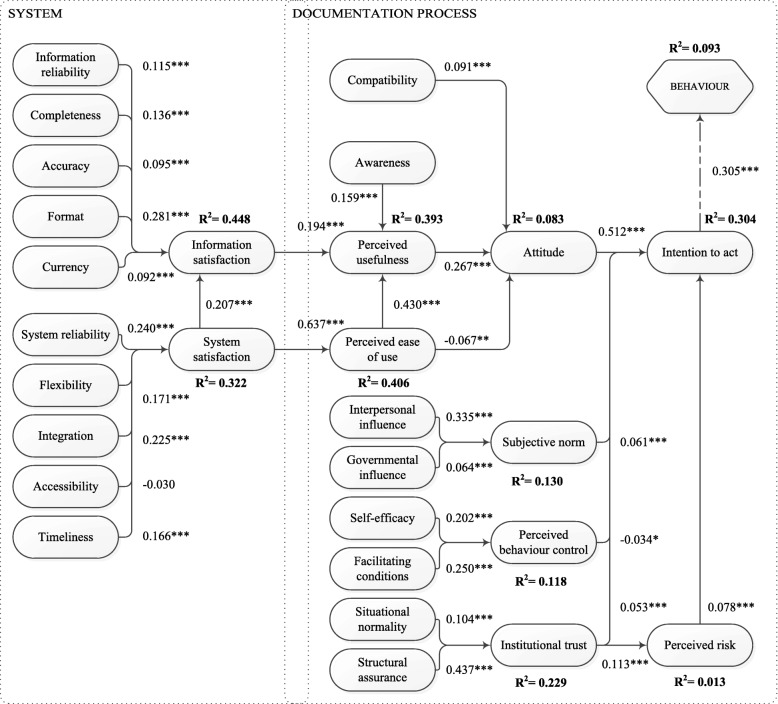
The (structural) model with path coefficients and coefficients of determination (R^2^). * *p* < 0.05, ** *p* < 0.01, *** *p* < 0.001

To validate our structural model, we evaluated the coefficients of determination (R^2^) and the size and relevance of the path coefficients. Figure 3 shows the resulting structural model with all the path coefficients and the coefficients of determination. All but three path coefficients were significant at *p* < 0.001. Only accessibility (*p* = 0.0531), perceived ease of use (*p* = 0.0012), and perceived behavioural control (*p* = 0.0202) had higher *p*-values. The coefficients of determination (R^2^) ranged from 0.013 (perceived risk) to 0.448 (information satisfaction).

To evaluate the impact of missing values in our dataset we repeated all tests on four additional imputed datasets. The results showed similar outcomes for all used validation measures (available from authors).

To investigate whether the performance of the model would be different based on only the data from medical specialists or nurses we did two additional validations using the data of only these subgroups. The results and distribution of performance measures of these two validations were comparable to the original measures (see Additional file 2 for the results). More importantly, when we apply the same target values for these additional validations as described in Table 1, the performance of the additional models is the same as that of our general model using all available data.

## Discussion

In this study, we constructed and validated a theoretical model representing underlying concepts that influence the adoption of structured and standardised data recording by healthcare professionals. The model includes concepts related to information systems as well as organisational factors and personal beliefs. The results of the model validation give credence to the model’s concepts and interrelationships. Additional validation of two models based on subsets of the respondents (either medical specialists or nurses) show comparable performance of these models.

First we validated the measurement model showing whether our questions (from the questionnaire) reliably measure the concepts (from our theoretical model). We found the measurement model had satisfactory composite reliability for exploratory models (i.e. models developing theory). The measurement model does satisfy the Fornell-Larcker criterion, which is a measure of discriminant validity. For six of our 12 relevant variables (i.e. blocks) the loadings of our observed variables are satisfactory for all items. For the other six variables, the loadings of one or more items were less than the required threshold, especially behaviour scores low in this respect. However, for all variables, at least one item scored above the threshold. The loadings indicate that a number of observed variables (i.e. questions from the questionnaire) could be removed from the model to improve both the efficiency of the questionnaire and the accuracy of the model. This could also improve the Average Variance Extracted (AVE) of the latent variables that are too low at this moment. The cross-loadings indicate a similar pattern that a small number of observed variables could be removed, most notably within the latent variable behaviour. In this study we performed a summative evaluation to validate our theoretical model. Future research should investigate the effect of model adaptations on the performance of the model.

Second, we validated the structural model, showing whether the variables of our theoretical model and their interrelations are valid. The validation shows that the R^2^ of the concepts are higher in the left part of the model. This is the part with concepts that have been developed and validated in multiple other studies (e.g. information and system satisfaction [13]). The lower scores are most prominent for attitude (0.083), behaviour (0.093), and perceived risk (0.013). These three concepts need further research to find the missing explaining underlying variables. The strongest indicator for intention to act is attitude. This means that it is important that the attitude of the healthcare professionals is positive with respect to structured and standardised data recording.

The only coefficient that was not significant at all was that of accessibility (to system satisfaction). The questionnaire was used in a high resource setting (the Netherlands) where the EHR and power are available 24/7. Therefore, although accessibility is not significant in our setting, it might become more significant in lower-resource settings. All other path coefficients were significant (*p* < 0.05 for perceived behavioural control and perceived ease of use) to very significant (*p* < 0.001).

A main strength of our study is that we based our model on existing and validated models. In particular, the underlying Technology Acceptance Model (TAM), commonly used outside [14] and within healthcare [15]. As Holden and Karsh state TAM “predicts a substantial portion of the use or acceptance of health IT” [15] however they also mention that the theory might benefit from additions and modifications [15]. Or, as concluded by Legris et al., it is has to be integrated into a broader model [14] as we did in this study. Another major strength is the large number of respondents to our questionnaire. This created a large sample size for our validation with structural equation modelling using the partial least squares method. By including healthcare professionals from seven out of eight different university hospitals we gathered data independent of the centre-specific context, such as the used documentation processes or EHRs.

A limitation of our study is the large proportion of missing data in our dataset. However, we used four additional imputed datasets in the analyses to evaluate the effect of imputing the missing data on the results of the validation. These analyses showed very similar results to the ones presented in this paper, thus justifying the robustness of the findings. Another limitation is that we cannot precisely calculate the response rate of our questionnaire since we do not definitively know who has received the email with the invitation to participate in our study.

If we compare our results with those from the two underlying models to our model [6, 7] we find that our model has lower coefficients of determination than the original models. The different focus, standardised and structured recording at the point of care, instead of system acceptance, and the different population (work field and country) could probably have attributed to this difference. Perceived risk is the lowest scoring concept and information satisfaction the highest matching concept in both our own model and the original source models.

Our questionnaire is based on self-reported outcomes and intentions. Further research will have to measure the exact compliance of healthcare professionals to structured and standardised data recording. When self-reported outcomes can be compared to the actual uptake of structured and standardised data recording we can evaluate whether the respondents are capable of a good assessment of their own compliance.

## Conclusions

First and foremost, our model helps to further understand the barriers and facilitators for healthcare professionals to adopt structured and standardised data recording. Additionally, our model and accompanying questionnaire can be used by hospitals to measure their own adoption and progress over time. When measuring in multiple centres, the results can be used to benchmark the scores and to identify best practice hospitals. Based on what best performing centres do differently, other hospitals can consider to adopt promising practices to improve their own adoption. Repeating the measurement at some other time in the future may indicate whether the changes have had effect on the adoption.

## Additional files

Additional file 1: English translation of used questionnaire (PDF 111 kb)

Additional file 2: Three additional tables showing 1) The percentage of missing data from each question in the questionnaire, 2) the cross loadings of the model, and 3) the Fornell-Larcker criterion. Also contains the performance measures of the two validations based on subgroups of our respondents (either medical specialists or nurses). (XLSX 125 kb)

## Abbreviations

AVE: Average Variance Extracted
EHR: Electronic Health Records
PLS: Partial Least Squares
SEM: Structural Equation Modelling
TAM: Technology Acceptance Model
