# Supplementary material for: Development and validation of a model for the adoption of structured and standardised data recording among healthcare professionals
Source: BMC Med Inform Decis Mak. 2018 Jun 28;18:54. doi: 10.1186/s12911-018-0640-8 (PMC6027789; doi:10.1186/s12911-018-0640-8)
Supplement: Supplementary file 1 — English translation of used questionnaire (PDF 111 kb) [file 12911_2018_640_MOESM1_ESM.pdf]

InformationReliability1	I trust data that a colleague recorded
InformationReliability2	I can trust data that a patient has recorded
InformationReliability3	I trust data that I recorded myself
InformationReliability4	When I change data, I trust that the new data is updated throughout the entire patient record
Completeness1	The patient record contains all the information I need
Accuracy1	The data in the patient record represent reality
Accuracy2	The data in the patient record contain few errors
Format1	The patient record has a clear interface
Format2	Because of clear design the data in the patient record can easily be recognised
Currency1	The data in the patient record are up to date
InformationSatisfaction1	The data in the patient record meet my expectations
SystemReliability1	I can trust that the EHR functions
Flexibility1	In different situations I can use the patient record flexible in my own way
Integration1	I have to use different computer programs to gather all patient data
Integration2	The patient record brings together data that used to be in various places
Accessibility1	I can access the patient data at any desired location
Timeliness1	The EHR responds fast enough to my orders
SystemQuality1	Our organisation has a high quality EHR
Compatibility1	The patient record supports my personal work processes
Awareness1	I know for what purposes the data that I record can be used other than providing care
Awareness2	I understand that data have to be recorded structured and standardised
Awareness3	I know how to record data to enable reuse (e.g. for discharge letters and in research)
PerceivedUsefulness1	When referring a patient to a care provider outside of the hospital I can easily and timely send all required information
PerceivedUsefulness2	The patient record ensures that all care professionals around a patient are well-informed
PerceivedUsefulness3	The patient record facilitates agreement with colleagues on the treatment plan of the patient
PerceivedUsefulness4	The patient record helps me to provide good quality patient care
PerceivedUsefulness5	Recording data in a structured and standardised manner costs me more time than recording in free text
PerceivedEaseOfUse1	I can always find the patient data that I need in the patient record
PerceivedEaseOfUse2	The patient record is user friendly
PerceivedEaseOfUse3	The patient record makes it easy to record data in a structured and standardised manner

Attitude1	I like recording data in free text
Attitude2	I like working in a structured and standardised manner
Attitude3	It is important that patient data can also be used by managers and researchers
Attitude4	It is important to record patient data directly at the point of care
Interpersonal1	My supervisor stimulates me to register data in a structured and standardised manner
Governmental1	External organisations like the inspectorate emphasize that I should record structured and standardised
SubjectiveNorm1	I record data in a structured and standardised manner because my colleagues expect it from me
SelfEfficacy1	I properly mastered working with the patient record
FacilitatingConditions1	There is enough time to properly record patient data
PerceivedBehavioural1	I can control whether the patient data is properly recorded in the patient record
SituationalNormality1	In my organisation proper data recording goes without saying
StructuralAssurance1	My organisation makes sure the patient record always functions
StructuralAssurance2	My organisation makes sure that patient data cannot be accessed by unauthorised persons
InstitutionalTrust1	I trust that my organisation manages the patient record safely
PerceivedRisk1	Reuse of data I recorded can harm the privacy of the patient
PerceivedRisk2	Reuse of patient data can lead to errors in the care provision
IntentionToAct1	I want to reuse as much available data as possible
IntentionToAct2	I want to record data structured and standardised
Behaviour1	I record many data twice or more (in multiple systems)
Behaviour2	I record as many data structured and standardised
Behaviour3	I reuse as many available data as possible
Behaviour4	I record data in such a way that others can use and reuse my data
Behaviour5	I register an allergy structured and standardised in the patient record as soon as there is new information
Behaviour6	I record medication structured and standardised in the patient record when there is new information
Behaviour7	I record all diagnoses using a standard list instead of in free text
Behaviour8	I record all procedures using a standard list instead of in free text
Behaviour9	I record a pain score structured and standardised in the patient record when there is new information
Behaviour10	I record vital parameters (e.g. pulse, blood pressure, respiratory rate, conscience) structured and standardised when there is new information
Behaviour11	I record risk of falling structured and standardised in the patient record when there is new information
